# Process evaluation of APPLE-Tree (active prevention in people at risk of dementia through lifestyle behaviour change and technology to build resilience): dementia prevention study focused on health and lifestyle changes

**DOI:** 10.1192/bjo.2025.10874

**Published:** 2025-11-21

**Authors:** Elenyd Whitfield, Claudia Cooper, Harriet Demnitz-King, Sedigheh Zabihi, Julie A. Barber, Mariam Adeleke, Rachel M. Morse, Amaani Ahmed, Alexandra Burton, Iain Lang, Penny Rapaport, Anna Betz, Zuzana Walker, Jonathan Huntley, Helen C. Kales, Henry Brodaty, Karen Ritchie, Elisa Aguirre, Michaela Poppe, Sarah Morgan-Trimmer

**Affiliations:** Wolfson Institute of Population Health, Queen Mary University of London, London, UK; Department of Statistical Science, University College London, London, UK; College of Medicine and Health, University of Exeter, Exeter, UK; Division of Psychiatry, University College London, London, UK; Department of Psychiatry and Behavioral Sciences, University of California, Sacremento, CA, USA; School of Psychiatry, University of New South Wales, Sydney, Australia; French National Institute of Medical Research, Paris, France; European University, Madrid, Spain; Department of Health and Community Sciences, University of Exeter, Exeter, UK

**Keywords:** Dementias/neurodegenerative diseases, process evaluation, cognitive impairment, intervention, dementia prevention

## Abstract

**Background:**

This concurrent, exploratory, mixed-methods process evaluation, embedded within a randomised controlled trial, investigates how the ‘active prevention in people at risk of dementia through lifestyle behaviour change and technology to build resilience’ (APPLE-Tree) secondary dementia prevention intervention might support behavioural and lifestyle goal attainment, through determining the contexts influencing engagement and testing intervention theoretical assumptions.

**Aims:**

We aimed to investigate (a) intervention reach, dose and fidelity, (b) contexts influencing engagement and (c) alignment of findings with theoretical assumptions about how the intervention might have supported participants to meet personalised behavioural and lifestyle goals.

**Method:**

We measured intervention reach and dose. We selected interviewees for setting, gender and ethnic diversity from the 374 APPLE-Tree trial participants randomised to the intervention arm. We interviewed 25 intervention participants, 12 facilitators and 3 study partners. Additionally, we analysed 11 interviews previously conducted during or after intervention delivery for an ethnography, and 233 facilitator-completed participant goal records. We thematically analysed data, combining inductive/deductive approaches informed by the ‘capability, opportunity and motivation-behaviour’ (COM-B) behaviour change model. We video-recorded a randomly selected tenth of sessions and rated fidelity.

**Results:**

A total of 346 of 374 (92.5%) intervention arm participants received some intervention (reach), and 305 of 374 (81.6%) attended ≥5 main sessions (predefined as adhering: dose). According to facilitator records, participants met a mean of 5.1 of 7.5 (68.3%) goals set. We generated three themes around (a) building capability and motivation, (b) connecting with other participants and facilitators and (c) flexibility and a tailored approach.

**Conclusions:**

The intervention supported behaviour change, through increasing knowledge and providing space to plan, implement and evaluate new strategies and make social connections. Feedback indicated that the intervention was flexible and inclusive of diverse preferences and needs.

The number of people with dementia is expected to increase to 153 million worldwide by 2050.^1^ Many dementia risk factors are potentially modifiable, including cardiometabolic factors, physical inactivity and social isolation.^2^ Nearly half of individuals who consult primary care with memory loss symptoms develop dementia within 3 years.^3^ While many receive lifestyle advice, there is limited evidence to support specific interventions.^4,5^ Compelling evidence for the impact of lifestyle or psychosocial interventions on cognitive decline in people at increased dementia risk comes from the Finnish Geriatric Intervention Study to Prevent Cognitive Impairment and Disability (FINGER), a randomised controlled trial (RCT) involving a 2-year intensive intervention delivered by experts, promoting nutrition, exercise, social connections, cognitive training and vascular risk management.^6^

It is estimated that delaying Alzheimer’s disease onset by 1 year will reduce the number of cases in 2050 by 11% worldwide,^7^ so the potential benefit of secondary dementia prevention, which aims to mitigate risk factors and promote brain health in people who are at risk of developing dementia, is considerable. Such interventions need to be widely acceptable, deliverable at scale and inclusive of those experiencing socioeconomic deprivation and other, including ethnic, minority groups at increased dementia risk.^8,9^

The ‘active prevention in people at risk of dementia through lifestyle behaviour change and technology to build resilience’ (APPLE-Tree) dementia prevention intervention was co-designed to be acceptable, inclusive and delivered at scale. We recruited participants between 2020 and 2022; the logic model^10^ (see Appendix A in the Supplementary Material available at https://doi.org/10.1192/bjo.2025.10874) and a pilot study^11^ are reported. In this process evaluation, conducted before knowing the trial results^12^, we aimed to investigate (a) intervention reach, dose and fidelity, (b) contexts influencing engagement and (c) alignment of findings with theoretical assumptions about how the intervention might have supported participants to meet personalised behavioural and lifestyle goals.

## Method

### Study design

We used the Medical Research Council (MRC) guidance on evaluating complex interventions.^13^ Based on the design and development of the logic model,^10^ we selected a theory-driven, mixed-methods approach. The London (Camden and Kings Cross) Research Ethics Committee (reference no. 19/LO/0260) and the UK Health Research Authority approved this study in April 2019. The protocol is registered (no. ISRCTN17325135). We designed the sampling strategy and topic guides to test *a priori* theories about how the intervention works, and causal assumptions regarding intervention mechanisms developed during co-design and iterated by the research and Patient and Public Involvement (PPI) groups.

### Intervention description

APPLE-Tree was delivered to groups of four to nine participants. The intervention aimed to promote healthy lifestyle, increase pleasurable activities and social connections and improve the long-term condition self-management. Facilitators delivered ten 1 h group video-call sessions over 6 months (fortnightly), alternating with ten video-call ‘tea-breaks’ (less structured, facilitated social sessions); facilitators conducted individual goal-setting telephone calls fortnightly, to a SMART ‘specific, measurable, achievable, relevant and time-bound’ (SMART) guide. They followed a script, but with flexibility to support goal-setting and review goal progress, troubleshooting barriers. The goal-setting book was co-designed by the co-production group, and held by participants to record their goals and monitor progress.

Full adherence was defined *a priori* as having received at least five main sessions. From months 6 to 12, participants met monthly to discuss how they were implementing learnt strategies, with those not attending receiving monthly goal-setting telephone calls or, if they preferred, discussion by email. Participants had access to a website with cognitive training, and received a single food delivery and a pedometer.

The intervention was delivered by 16 university-employed facilitators, psychology or social science graduates, paired with 15 third-sector or National Health Service (NHS)-employed facilitators (psychology assistants, who do not have formal clinical training); or at two sites, by two NHS-employed facilitators. All facilitators (31 in total) were non-clinically trained. They attended training and group supervision fortnightly with a clinical psychologist and monthly with a nutritionist (A. Betz). Further intervention details are published elsewhere.^10^ A ‘template for intervention description and replication’ (TIDIER) is shown in the Supplementary Material Appendix B.

### Setting and sample

The APPLE-Tree trial included 746 people aged 60+ years with either subjective cognitive disorder (SCD) or mild cognitive impairment (MCI) defined using Quick Mild Cognitive Impairment-published thresholds,^14^ without dementia, recruited via mail-outs from participating general practices, memory clinics and social and print media. Further details are published elsewhere.^10^ Participants were randomised using 1:1 allocation to intervention or control arms (provision of dementia prevention information).

Of 746 trial participants, 374 were randomised to the intervention arm. Inclusion criteria for the process evaluation interviews were (a) being an intervention participant (or having withdrawn from the intervention and not the study); (b) study partner (family member or friend) of a participant; or (c) an intervention facilitator. We initially approached all participants, study partners and facilitators from four intervention groups (group refers to cohorts of four to nine participants who attended the same group sessions), purposively selected to encompass urban and rural, NHS and non-NHS contexts. We recruited additional participants from minority ethnic groups and female participants, from across intervention groups, to increase sample diversity. The primary outcome for the main trial was neuropsychological test battery score at 24 months.

Sample size for the process evaluation interviews was planned (estimated *a priori* as 45 in total) to ensure sufficient diversity for setting (urban/rural, NHS/non-NHS), gender and ethnic diversity.^16^ Process evaluation participants gave written or audio-recorded informed consent.

In addition to the primary interviews, secondary analysis was carried out on 11 interviews conducted with APPLE-Tree intervention arm participants during and after the intervention in an embedded visual ethnography study.^17^

### Data collection

Qualitative interviews were conducted following completion of the main intervention groups (except for two participants interviewed after session seven and one after session six, who volunteered and were interviewed earlier for logistical reasons) by researchers not involved in intervention delivery or outcome assessment. Interviewees who withdrew from the intervention were interviewed within 2 months of this decision. Topic guides (Supplementary Material Appendix C) drew on the logic model (Supplementary Material Appendix A)^10^ to explore how participants had experienced the intervention, were supported to make and maintain lifestyle changes and perceived the impact of these changes. E.W. (social science researcher), research assistants and a clinical psychologist conducted interviews between September 2021 and May 2023.

Additional data analysed: E.W. reviewed transcripts of 11 participant interviews from a visual ethnography conducted during the intervention (March–September 2022) to identify data potentially relevant to our aims, which were included in this analysis.^16^ These were intervention participants who engaged in a separate study using photographs, interviews, workshops and an exhibition to explore their lived experiences. The interviews included some questions about experiences of the intervention. The interviews were coded thematically in NVivo alongside interviews conducted specifically for the process evaluation.

Facilitators recorded goals set, their content and whether they were achieved using a standardised spreadsheet, on which they made written notes during or immediately after goal calls. We randomly selected (using random number generation) one of the 10 main intervention sessions from each intervention group for video-recording (*n* = 41) to assess facilitator adherence to procedures, using a standard fidelity checklist approach similar to one developed for a previous intervention^17^ (see Supplementary Material Appendix D). Information on participants’ use of cognitive training was downloaded from the study website.

### Analysis

We compared the trial and process evaluation samples with each other, with the 2021 census data for England and Wales^18^ and with the English Housing survey.^19^ We described intervention reach, adherence and fidelity using summary statistics. We analysed goal content at participant level, reporting numbers of goals that corresponded with goal themes, generated through content analysis. We thematically analysed the interviews. Interviews were coded in NVivo by E.W., both deductively and inductively, drawing on COM-B model concepts.^21^ In this model, each modifiable factor (capability, opportunity, motivation leading to a behaviour) comprised two parts. Psychological capability includes knowledge and understanding; physical capability refers to physical attributes and bodily capacities; reflective motivation to conscious processes such as evaluation and planning; automatic motivation to desires, habits and impulses; and opportunity to the physical and social contexts of behaviours.^21^ E.W. developed codes corresponding to these concepts as nodes in NVivo version 12 for Windows (Lumivero, Denver, CO, USA; https://lumivero.com/products/nvivo/), developing other codes inductively and grouping nodes. The codebook and three de-identified transcripts were discussed with co-authors C.C., S.M.-T., R.M.M. and A.A. In a minor deviation from the protocol, our analysis working group analysed the same three transcripts, to enable richer discussion, rather than as originally planned, for two members to separately code four (10%) of the transcripts. E.W. continued to engage with the data, to support the development of themes.

A clinical psychologist listened to intervention session recordings, completing fidelity checklists. We calculated the proportion of expected intervention components delivered using the fidelity checklist. We rated fidelity according to established thresholds, with 81–100% constituting high fidelity. On a 5-point scale (1 – not at all to 5 – very much) we rated whether facilitators kept the group focused, participants engaged in each intervention component and the session kept to time.

We collected and analysed quantitative and qualitative data using the method of concurrent triangulation. Qualitative interview data were contextualised by attendance and fidelity data.

The authors assert that all procedures contributing to this work comply with the ethical standards of the relevant national and institutional committees on human experimentation, and with the Helsinki Declaration of 1975 as revised in 2013.

## Results

### Quantitative findings

#### Reach and dose

Compared with census data for people aged 65 years and above, trial and process evaluation samples included more people from non-White ethnic groups (11.6 and 13.9%, respectively, relative to 6.4% in the census) and those living as a couple (66.5 and 63.9 *v*. 60.7% in the census).^18^ While 80% of people in England aged 65+ are home owner-occupiers, this was higher in trial (88.0%) and process evaluation (83.3%) samples.^19^

Among 374 intervention arm participants, 346 (92.5%) received 1 or more main intervention sessions, 305 (81.6%) attended 5 or more main sessions and 89 (23.8%) attended all 10 main sessions; 2363 of 3279 (72.1%) possible tea-breaks and 983 of 1903 (51.7%) implementation catch-ups, scheduled between 6 and 12 months, occurred. Of the 374 participants, 57 (15.2%) withdrew from the intervention; 49 (13.1%) intervention arm participants used the cognitive training app, 13 (3.5%) accessed the intervention using a device provided by the study team while all others used their own devices. The cognitive training app was specially designed by a student in the university.

#### Fidelity

Mean rater fidelity scores out of 5 were: 4.69 for ‘Keeping the group focused on the manual’, 4.51 for ‘Keeping participants engaged’ and 4.97 for ‘Keeping the session to time’. Overall, fidelity was high (94.5%, 14.17/15).

#### Goal data

We had usable goal-setting call data for 233 participants. Data on numbers of goal calls and goals set were available for 208 participants, who set between 0 and 23 goals and achieved an average of 5.1/7.5 (68.3%) goals set in the first 6 months of the intervention. The main reason for missing goal data was external (third-sector) facilitators not providing them to the study team.

Records of goal content were available for 228 participants. Participants made goals related to: improving diet (*n* = 203); increasing exercise (*n* = 189); relaxation, mood and well-being (*n* = 90); planning and reflecting on activities (*n* = 79); improving physical health (*n* = 77); sleeping better (*n* = 64); engaging with valued activities (*n* = 64), including creative and artistic activities (*n* = 24); cognitive stimulation (*n* = 51); connecting with others (*n* = 47); engaging with the programme (*n* = 25); reducing alcohol intake (*n* = 24); reducing smoking (*n* = 2); completing other tasks (*n* = 18); managing memory issues (*n* = 11); and reducing screen time (*n* = 6).

### Interview sample description

Forty participants were included in the sample: 19 who completed the intervention and 6 who withdrew: 1 withdrawer rejoined and also completed the programme. Goal data were available for 10/20 and 3/5, respectively. The sample also included 12 facilitators and 3 study partners: 2 daughters and 1 wife of a participant, all of White UK ethnicity. Additionally in the analysis we included 11 interviews from a previous visual ethnography, which is a research approach using visual methods – in this instance photographs in addition to interviews – to explore their lived experiences.^16^

Table 1 shows that, relative to the baseline trial population, those interviewed for process evaluation were more likely to be female and from a non-White UK ethnic group. Table 2 summarises process evaluation data.

**Table 1:** Characteristics of the interview sample compared with baseline trial population

Characteristics	All (*N* = 746)	Interview sample (*n* = 36)
Age, mean (s.d.), range	74.4, (6.9) **(*n* = 742)**, 60.1–102.7	74.6 (6.3) **(*n* = 36)**, 61.6–90.6
Gender, *n* (%)	**743**	**36**
** **Male	392 (52.8)	16 (44.4)
** **Female	350 (47.1)	20 (55.6)
** **Other	1 (0.1)	0 (0)
Ethnicity, *n* (%)	**742**	**36**
** **White UK	599 (80.7)	28 (77.8)
** **White other	57 (7.7)	3 (8.3)
** **Asian	50 (6.7)	2 (5.6)
** **Black	13 (1.8)	2 (5.6)
** **Mixed	16 (2.2)	1 (2.8)
** **Arab	3 (0.4)	0 (0)
** **Other	4 (0.5)	0 (0)
Marital status, *n* (%)	**742**	**36**
** **Single	46 (6.2)	1 (2.8)
** **Married/civil partnership	464 (62.5)	21 (58.3)
** **Living with partner	30 (4.0)	2 (5.6)
** **Widowed	108 (14.6)	8 (22.2)
** **Divorced	92 (12.4)	3 (8.3)
** **Unable to specify	2 (0.3)	1 (2.8)
Highest level of education, *n* (%)	**742**	**36**
** **No education	2 (0.3)	0 (0)
** **Primary	10 (1.3)	1 (2.8)
** **Secondary (e.g. O-level, GCSE)	167 (22.5)	11 (30.6)
** **Further (e.g. A-level, BTEC, NVQ)	199 (26.8)	7 (19.4)
** **Degree	204 (27.5)	7 (19.4)
** **Postgraduate	147 (19.8)	10 (27.8)
** **Other	11 (1.5)	0 (0)
** **Unable to specify	2 (0.3)	0 (0)
Employment, *n* (%)	**742**	**36**
** **Full-time employment	32 (4.3)	2 (5.6)
** **Part-time employment	54 (7.3)	1 (2.8)
** **Retired	607 (81.8)	31 (86.1)
** **Unemployed/unable to work	17 (2.3)	0 (0)
** **Other	29 (3.9)	2 (5.6)
** **Unable to specify	3 (0.4)	0 (0)
Living situation, *n* (%)	**742**	**36**
** **Living alone	198 (26.7)	10 (27.8)
** **Living with other relative(s)	532 (71.7)	26 (72.2)
** **Living with friend(s)/other people	6 (0.8)	0 (0)
** **Other	6 (0.8)	0 (0)
Type of accommodation, *n* (%)	**742**	**36**
** **Council rented	33 (4.4)	2 (5.6)
** **Privately rented	40 (5.4)	2 (5.6)
** **Own home	653 (88.0)	30 (83.3)
** **Supported living	12 (1.6)	2 (5.6)
** **Other	4 (0.5)	0 (0)
Diagnosis, *n* (%)	**742**	**36**
** **Mild cognitive impairment	125 (16.8)	8 (22.2)
** **Memory concerns or problems	59 (8.0)	2 (5.6)
** **Other	13 (1.8)	0 (0)
** **Not given diagnosis	525 (70.8)	25 (69.4)
** **Unable to specify	20 (2.7)	1 (2.8)

GCSE, General Certificate of Secondary Education; BTEC, Business and Technology Education Council; NVQ, National Vocational Qualification.

Sample sizes are in bold.

**Table 2:** Description of process evaluation data, by group

						Additional primary interviews	Secondary interviews analysed
Participants’ data	Group 1 (NHS, suburban), *n* = 3	Group 2 (non-NHS, rural), *n* = 3	Group 3 (non-NHS, urban/suburban), *n* = 3	Group 4 (non-NHS, urban/suburban), *n* = 3	Participants (withdrew), *n* = 6	Group 5 (recruited from multiple intervention groups), *n* = 7	*n* = 11
Participants (completed intervention)	3	3	3	3	1 (1 rejoined following the interview)	7	11
Goal data available	3	2	2	0	3	3	7
Gender	75% male, 25% female, 0% other	100% male, 0% female, 0% other	100% male, 0% female, 0% other	33.3% male, 66.6% female, 0% other	16.6% male, 83.3%, female, 0% other	42.8% male, 57.1% female, 0% other	27.2% male, 72.7% female, 0% other
Ethnicity, %							
** **White UK	100	100	100	66.6	100	71.4	54.5
** **White other	0	0	0	0	0	14.2	18.1
** **Asian	0	0	0	0	0	14.2	9
** **Black	0	0	0	33.3	0	0	9
** **Mixed/other	0	0	0	0	0	0	9

NHS, National Health Service.

### Qualitative findings

#### Theme 1: building capability and motivation: increased capability and reflective motivation altered automatic motivation

The intervention increased capability through supporting learning, and reflective motivation by facilitating evaluation and planning. Learning was primarily through main group sessions and an accompanying course book. Evaluation and planning were facilitated through goal-setting. Discussions of ideas, plans and achievements took place in group sessions and tea-breaks, with ‘modelling’ behaviours as a ‘motivational device’.^20^ In the subthemes below, we describe how the intervention increased (a) capability, (b) reflective motivation and (c) automatic motivation. The content of goal call sheets supports findings from interviews because they illustrate that, for some participants, one-to-one discussions with facilitators supported them in working towards lifestyle changes.

#### Subtheme 1: increased capability

Participants learned new information about healthy behaviours and the science of familiar healthy messages. Some participants and facilitators described how the intervention reinforced familiar messages. Others, including some who withdrew, felt that information was insufficiently novel; one facilitator described how they responded to this:

‘“Well, I already know that” and that’s if you leave it at the content level as you already know. If you take it to a meta level, “Yes, we already know that” but it’s “This is why it’s relevant when seen through the APPLE-Tree lens, why it’s important, and thinking about MCI [mild cognitive impairment], brain health”.’ (facilitator, NHS Trust)

New information was described by them as ‘a way of giving that control’, prompting reflective motivation around health improvements: ‘how do I look at what I do and decide is that good for me?’

Goal calls were an opportunity to explore barriers to healthy habits:

‘It was helpful to then use the goal calls to work through what barriers, why aren’t they doing it, why would they do it? And do it that way.’ (facilitator, research assistant)

Among people who withdrew from the intervention, disjuncture between expectations and course content was a common theme. They expressed surprise that there was not more focus on cognition and memory, as did some participants who completed the course. One participant was motivated to make lifestyle changes, but felt that her physical health problems and living alone prevented engagement with the suggested strategies.

#### Subtheme 2: reflective motivation

New information was described as ‘a way of giving that control’, prompting reflective motivation around health improvements: ‘how do I look at what I do and decide is that good for me?’ (facilitator, NHS Trust). Participants discussed changes (evaluating and planning) in group sessions and one to one in the goal calls.

One facilitator described ‘little epiphanies’ when people tried new activities. A participant interviewed between sessions 6 and 7 reflected on the difference between knowing what to do and taking action. She spoke of how the programme was helping her to develop better habits:

‘Being aware of it and putting it into your daily routine are two different things, and this study has made, how shall I put it, made me take responsibility … I have started introducing the changes in my diet and lifestyle … this is helping because every week having this check in, the tea-breaks and all the things, so it is kind of helping me to form that habit.’ (participant, group 5, female, aged 70–80 years)

She also faced her fear of swimming following a traumatic incident, describing it as ‘a very big step’.

Increasing step count was a popular goal. One participant wore the pedometer in bed to include steps at night, saying that it is ‘part of me now’ (participant, secondary analysis, female, aged 60–70 years). An alcohol unit calculator included in one session was noted by a facilitator, to allow people to take ‘more power back over better decisions and their choices’ (facilitator, research assistant). Few intervention arm participants (*n* = 49) used the cognitive training app, many citing technical barriers.

#### Subtheme 3: automatic motivation

These increases in capability and reflective motivation enabled changes in habits (automatic motivation). This included eating more nuts, less sugar and more olive oil, adopting a ‘brain-healthy’ diet, taking supplements, reading food labels, reducing alcohol intake, increasing exercise, drinking more water, checking blood pressure and joining new social activities. Some participants reported health benefits that in turn increased capability, such as weight loss, more energy, stamina and improved sleep; one participant reported reversing their pre-diabetes. These changes align with our *a priori* logic model.

Some participants were encouraged to return to activities that they had previously discontinued,

‘I took on the Tai chi … that was good. I used to do it a few years back, but then I had a bit of a balance problem and couldn’t do it, so I let it drop. Now I’ve started again on that, and so far, touch wood, it’s all right.’ (participant, group 2, male, aged 80–90 years)

A participant who had had a hip replacement and knee operation gained motivation to recommence jogging:

‘I think that motivation saying, “Look, you can do it”, was very good for me. It just got me off my backside and said, “Come on, you’ve got to jolly well do it”.’ (participant, group 3, male, aged 70–80 years)

Some participants described changes in outlook, increased confidence and self-efficacy and empowerment, reflecting increased psychological capability and altered habits of thought and action. In this next example, a participant described reflecting on how his daily activities affected him:

‘How did my mood or my mentality change because of the adaptation of what happened? So it’s a lot of things. Instead of looking at things blinkered, I now look at things in a very wide sphere.’ (participant, group 2, male, aged 60–70 years)

For some, the intervention was empowering and enabled changes in routines. One study partner felt that their father was more open to new ideas now and taking greater responsibility for his own health, suggesting an increased agency and capability, which were reflected in changes to his daily habits:

‘… he is quite happy to go to the supermarket now. Something as basic as that, whereas before my mum would do it, but he’ll quite happily go himself now … He’ll make his own doctor’s appointments rather than my mum doing it. So, there is a few things that he does do himself now which he didn’t do six months ago.’ (study partner of father in intervention, aged 60–70 years).

One facilitator felt that the intervention had been ‘fantastic for [participants with caring responsibilities] in reclaiming their autonomy in the habits of their daily lives; it helped them to evaluate and reflect on how they spent their time, and to make more time for themselves’. (facilitator, third sector). The activity diary was noted as being important in this process.

One participant described the impact of conversations with a facilitator in creating motivation and supporting changes in his eating habits:

‘She’s very skilled and steering, and you can see – you can feel yourself being nudged and steered. So she’s very good.’ (group 5, male, aged 70–80 years)

Another facilitator described the changes made by participants, from ‘massive’ to ‘really small’, but how even these could be ‘such a meaningful change’ and very beneficial.

The role played by participants’ social context in dietary changes was variable. Some partners actively supported positive dietary changes; one felt that she had ‘sort of been in partnership’ and was very pleased at the changes her partner made (study partner, group 2 participant’s wife, husband aged 70–80 years). However, habits and tastes of partners could also be a barrier to possible changes because shopping, cooking and eating are often shared, habitual activities.

#### Theme 2: connecting with other participants and facilitators helped increase social opportunity, motivation and capability

The APPLE-Tree groups created connections with others, motivating people to engage with the intervention and institute behaviour change:

‘One of the big positives for me that it was in a group environment rather than one on one. It’s nice to be able to share ideas and listen to other people’s experiences and to share your own ideas and experiences obviously. You don’t feel so isolated.’ (participant, group 5, male, aged 60–70 years)

One participant described how sharing experiences in the groups helped him realise that he was not ‘the only one’ experiencing memory problems:

‘We’re all in the same boat. But you don’t realise until you actually discuss it amongst yourselves, yeah. You just button it up.’ (participant, group 1, male, aged 60–70 years)

A facilitator gave examples of participants who, although initially sceptical about the programme, valued the social aspect:

‘Perhaps people who felt that their memory problems were quite isolating for them and felt quite a lot of frustration that perhaps their family didn’t understand what they were going through or felt embarrassed to talk about it, this was finally a space where they could talk openly about it, knowing that other people were experiencing similar things.’ (facilitator, research assistant)

Facilitators and participants described how some groups/group members met up in person or, in one case, in their own support group online after the programme; a few members of one group continued to meet at a local café:

‘It was quite a good mix of interests and abilities and I found I had things in common with several of them. And we are going to continue to meet together which is always a good thing.’ (participant, group 5, female, aged 80–90 years)

Positive relationships with group members motivated engagement:

‘It’s something I look forward to because it’s a change of scenery, it’s a different type of dialogue with different friends, so that’s a bonus as well.’ (participant, group 2, male, aged 60–70 years)

A facilitator expressed surprise at how important this social connection seemed to be:

‘The social element was often one of the main facilitators of the group and it would often be the things that meant people would come back each week because they felt they were benefiting from being kept accountable each week to the things they want to try or the things they want to change or they’re getting to know other people.’ (facilitator, research assistant)

Group discussions increased knowledge and motivation for behavioural change. A facilitator recounted how one participant’s goal of decluttering inspired others. Participants influenced each other to do online Tai chi, pilates and yoga. A participant described how he found hearing other people’s coping strategies ‘really encouraging’ (participant, group 3, male, aged 70–80 years).

Another described how sharing ideas motivated him:

‘Sharing those moments, sharing the different ideas, oh I didn’t think of that, that’s a good idea, I’m going to try that, is very, very useful.’ (participant, group 5, male, aged 60–70 years)

One participant’s partner described conversations in groups as ‘invaluable’ by sharing ‘You could say wow, she’s doing that. What could I do?’ (study partner, group 2, wife to participant aged 70–80 years). This again speaks to the reflective motivation that can be prompted through discussions.

Facilitators described how some groups ‘gelled’ more than others. One reflected on how, in her experience, this could inhibit levels of engagement:

‘Because they hadn’t gelled so much, it just made the group discussions a bit wooden. So there wasn’t so much involvement and then they weren’t super personable and that meant that the goals and other things that were … surface-level, but it felt like they weren’t engaging as much as they usually would have.’ (facilitator, third sector)

One participant felt that other group members were more physically capable than she was:

‘The exercise bits are very, very limited in what I can do. But there were little bits I could use and adapt. But they were the other end of the scale. You had people who were doing four hour fitness programmes either in the gym or walking for miles and miles every single day … And one of the effects it had on me was at the end of a session, I felt I had failed. And I mentioned this to one of the leaders in the individual call.’ (participant, group 5, female, aged 80–90 years)

One study partner described how their father enjoyed spending time with ‘completely different people from different walks of life’, outside of his usual ‘bubbles’. He had become more outgoing as a result: ‘He’s quite happy to sit with the other people who he doesn’t know, talk to people in the supermarket on the checkout and whatnot. So, he’s become a bit of a social butterfly.’ (study partner, group 1 participant’s daughter, father aged 60–70 years)

Group interactions in group 4 were reported positively by all participants, with three members meeting up in person. A study partner whose father was in group 2 described how it had ‘brought [him] out of his shell a bit more’, and he had ‘really enjoyed’ the meetings and tea-breaks (study partner, participant’s daughter, father aged 80–90 years).

Several participants who withdrew from the intervention because they found it unhelpful did not appear to have experienced this group cohesion; one stated that they were happy to talk one to one but not in a group. Many participants who completed the intervention spoke very positively about their facilitators’ encouragement and approach. One participant who felt less comfortable speaking in groups reflected on the ‘rapport’ they had built with the facilitator during goal calls: ‘I found I had no problem speaking quite openly. That I find a lot easier than doing it within the group.’ (participant, group 1, male, aged 70–80 years)

Good relationships enabled constructive goal setting, as described by a facilitator who reflected on the challenge of balancing relationship-building and goal-setting in one-to-one calls. Participants living alone seemed to particularly value these relationships:

‘There were some people at the table that live on their own in particular I ended up talking to a lot longer. They’d be 30 minutes, maybe even more, each time I had a goal call with them. But again, they were great, they set so many goals, it was perfect. But, again, I think there’s that fine line between having a chit-chat and setting goals. But it really helped to think about why they were doing it, and I think building a relationship made it better.’ (facilitator, research assistant)

Participants shared recipes, photographs and videos. Trying each other’s recipes built social connections. One participant, a yoga teacher, led a breathing exercise in a tea-break, intending to repeat this in online meetings after the group ended. Another participant shared their own meditation practice with the group.

#### Theme 3: a flexible, tailored approach increased capability, motivation and opportunity for engagement.

Facilitators tailored the intervention to individual needs and preferences, which helped motivate participants. Participants valued the choice provided by multiple intervention elements:

‘The flexibility of what participants take from the intervention and what they leave, because they’re not going to take everything … one of the main things as a facilitator … is to find their aspects of the intervention that work for them … let them lead on what they want to make changes in, and what aspects work for them.’ (facilitator, research assistant)

Another facilitator reflected that there is ‘something for everybody’ in the course content, which helped to facilitate motivation for behavioural change:

‘I think that’s what’s really good about APPLE-Tree is that in sessions one and two if there isn’t something for you there will be something for you in the later session to do with diet or exercise, that can kind of help with that. Or that will spark your interest and motivation, motivate you to make some changes.’ (facilitator, research assistant)

They described responding to resistance to lifestyle change by finding something else that a participant might value in the sessions, such as connecting with others. Another discussed ‘tweaking’ exercise videos to meet people’s needs, selecting lower-intensity options for those with reduced mobility (facilitator, third sector).

Tea-breaks were described as both ‘integral’ to the intervention by one facilitator, and as a flexible space that differed between groups by another:

‘Some groups really enjoyed the structure of recapping and talking about next week and … sharing recipe suggestions from other people in the groups. Then some of the tea-breaks were people just really connecting with each other.’ (facilitator, third sector)

They were opportunities for participants to take more of an active role:

‘I like the tea-breaks because you could discuss things, whereas in the sessions, you were sort of taught, and in the tea-breaks, you could talk about it.’ (participant, group 5, male, aged 70–80 years)

Groups discussed and shared cultural differences, enriching discussions. A participant from an African ethnic background described how he shared information about food from his culture (participant, group 4, male, age 70–80 years). Another participant discussed how she felt ‘very European’, and was surprised that another ‘very English lady’ was unfamiliar with courgettes (participant, group 5, female, age 70–80 years).

## Discussion

We describe how the APPLE-Tree intervention increased physical and psychological capability, through learning, with content delivered via group sessions and the course book. Reflective motivation was increased through planning and evaluation. Goal calls were the primary space for this, but group discussions were also important. The activity diary, included in the intervention booklet and discussed in sessions, was used for planning and reflective evaluation. Changes in habits improved capability further due to physical health improvements. Social connections alleviated a sense of isolation around memory issues for some, and motivated continued engagement through discussion and modelling.

Because groups were agents of change, their composition mattered. Groups valued the different experiences and diversity of memberships, supporting our aim to create inclusive spaces. This extends findings around the importance of social connection from the APPLE-Tree pilot study.^11^ By contrast, participants who found the intervention unhelpful did not experience groups as being cohesive or helpful; some felt unconvinced of a need to change lifestyle, or that the intervention did not equip them with personally relevant strategies. Reflections from facilitators, and goal call records, indicated that the programme offered flexibility to personalise the intervention, enabling positive change through diverse pathways. Intervention dose, reach and fidelity were high.

The APPLE-Tree intervention aimed to reduce cognitive decline, with secondary outcomes to reduce anxiety and depression and improve sleep, quality of life and functioning. Intended mechanisms include changes in diet, physical functioning, social networks and support.^10^ As hypothesised, the intervention improved cognition relative to the control condition.^1^ Some mechanisms hypothesised in the logic model are supported by the process evaluation, and by the main trial analysis, notably dietary change. Others are not: cognitive training was not widely taken up. Insights from this process evaluation, and from a recent pre-implementation study,^21^ can support larger-scale implementation.

‘The power of prevention’ is a focus of the Darzi independent investigation to inform the English NHS 10 Year Plan.^23^ Realising this ambition for secondary dementia prevention requires an acceptable, flexible intervention that supports personalisation. Delivery by non-clinical facilitators, including social prescribers, band 4 NHS workers and third-sector workers with skilled supervision,^24^ appeared to work well.^25^

We captured diverse experiences, including accounts from participants who withdrew. We used diverse methods, including ethnographic interviews, finding that this increased accessibility. For some participants with memory concerns, centring interviews around photographs evoked memories and the interviews provided opportunity for discussion of the intervention. There were some limitations. The intervention was delivered only in English within a well-resourced team. People living alone and in rented accommodation were underrepresented in this trial relative to the general population. In addition to structural barriers to research participation affecting people from socioeconomically deprived backgrounds in most trials,^26^ this may reflect their relative digital exclusion. All participants had memory concerns, so there may have been a recall bias towards more recent events. Use of contemporary goal call data partially mitigated this, although data were unavailable for 40% of participants. This could introduce bias, if external facilitators systematically differed in their goal-setting approaches to internal facilitators, although all received the same training. The trial took place during the pandemic and post-pandemic period: this context may have influenced how participants were able to use the intervention.^27^

The intervention was effective in facilitating capability, aided by psychoeducation and consequent learning and reflective motivation, promoting behaviour change and new habit formation. The group aspect and the promotion of group cohesion were important to this. Flexibility in the wide range of topics available, goal-setting approaches and the multiple components of the intervention enhanced its adaptability and potential usefulness.

## Supporting information

Whitfield et al. supplementary material 1 Whitfield et al. supplementary material

Whitfield et al. supplementary material 2 Whitfield et al. supplementary material

## Data Availability

The data that support the findings of this study are available on reasonable request from the corresponding author (E.W.). The data are not publicly available due to their containing information that could compromise the privacy of research participants.

## Supplementary material

The supplementary material is available online at https://doi.org/10.1192/bjo.2025.10874
