# Supplementary material for: Process evaluation of APPLE-Tree (active prevention in people at risk of dementia through lifestyle behaviour change and technology to build resilience): dementia prevention study focused on health and lifestyle changes
Source: BJPsych Open. 2025 Nov 21;11(6):e284. doi: 10.1192/bjo.2025.10874 (PMC12641417; doi:10.1192/bjo.2025.10874)
Supplement: Whitfield et al. supplementary material 1 — Whitfield et al. supplementary material [file S2056472425108740sup001.docx]

**Appendix A**

**APPLE-Tree logic model**

|  | **Appendix B**  **The TIDieR (Template for Intervention Description and Replication) Checklist:**  Information to include when describing an intervention and the location of the information  Item Primary Paper  (page or appendix number)    **BRIEF NAME** APPLE-Tree (Active Prevention in People Title  at risk of dementia through Lifestyle bEhaviour Change and  Technology to build REsiliEnce), |  |  |
| --- | --- | --- | --- |
|  |  |  |  |
|  | **WHY:** Dementia prevention Abstract |  |  |
|  |  |  |  |
|  | **WHAT:** we aimed to investigate how the APPLE-Tree secondary Abstract  dementia prevention intervention might support behavioural and  lifestyle goal attainment through determining contexts influencing  engagement and testing intervention theoretical assumptions. |  |  |
|  | Materials: Describe any physical or informational p. 2  materials used in the intervention, including those |  |  |
|  | provided to participants or used in intervention  delivery or in training of intervention providers. |  |  |
|  | Provide information on where the materials can be  accessed (e.g. online appendix, URL).  Course booklet (facilitator); course booklet, including diet quiz (participant); goal booklet; activity diary; 1 delivery of healthy food; pedometer; cognitive training app (<https://www.ucl.ac.uk/psychiatry/apple-tree-study/cognitive-training>); lifestyle change app. (<https://www.ucl.ac.uk/psychiatry/apple-tree-study/eden-app>). |  |  |
|  | Procedures: Describe each of the procedures, activities, and/or processes used in the intervention, | -  p.2 |  |
|  | including any enabling or support activities.  Main group sessions covering intervention material were run by two facilitators, plus support for accessing as the sessions are online. In addition, there were half hour informal ‘tea break’ sessions. After the first six months there were monthly, implementation, half hour group sessions for another six months. Individual goal setting ‘goal calls’ between facilitators and participants. |  |  |
|  | **WHO PROVIDED** |  |  |
|  | For each category of intervention provider (e.g. psychologist, nursing assistant), describe their | p. 2 |  |
|  | expertise, background and any specific training given.  The intervention was delivered by university-employed facilitators, psychology or social science graduates, paired with third sector or NHS-employed facilitators; or at two sites, by two NHS-employed facilitators. All facilitators were non-clinically trained. They attended training and group supervision fortnightly with a clinical psychologist (SB) and monthly with a nutritionist (ABe). A trained nutritionist provided support by answering questions and giving information to participants via facilitators. |  |  |
|  | **HOW** |  |  |
|  | Describe the modes of delivery (e.g. face-to-face or by some other mechanism, such as internet or | p. 2 |  |
|  | telephone) of the intervention and whether it was provided individually or in a group.  All group sessions were online. Individual goal calls were by telephone. |  |  |
|  | **WHERE** |  |  |
|  | Describe the type(s) of location(s) where the intervention occurred, including any necessary | p. 2 |  |
|  | infrastructure or relevant features.  The intervention was all delivered remotely, by video call and telephone. |  |  |
| **WHEN and HOW MUCH** | | | |
| Describe the number of times the intervention was delivered and over what period of time including  the number of sessions, their schedule, and their duration, intensity or dose. Abstract + p. 2  410 main sessions were delivered.  First six months: main group sessions and half hour informal tea break sessions were fortnightly,  alternating weekly, for six months. Individual ‘goal calls’ were fortnightly.  Following six months: Monthly implementation group sessions. | | | |
|  | | | |
| **TAILORING**  If the intervention was planned to be personalised, titrated or adapted, then describe what, why  when, and how. p.p. 5 + 10-11  One to one discussions (in individual goal calls) allowed tailoring and personalisation. Participants  worked with facilitators to set and work towards individual goals. A nutritionist was available to  answer specific questions and offer advice through facilitators. A range of topics were covered by the  intervention and the ‘tea break’ sessions could be used flexibly in response to the needs of different groups. | | | |
| **MODIFICATIONS**  If the intervention was modified during the course of the study, describe the changes (what, why,  when, and how). N/A  There were no modifications. | | | |
| **HOW WELL**    Planned: If intervention adherence or fidelity was assessed, describe how and by whom, and if any  strategies were used to maintain or improve fidelity, describe them. p. 3  41 individual sessions were video-recorded and reviewed by a clinical psychologist.  Actual: If intervention adherence or fidelity was assessed, describe the extent to which the p. 4  intervention was delivered as planned.  Mean rater fidelity scores out of five were: 4.69 for ‘Keeping the group focused on the manual’, 4.51 for  ‘Keeping participants engaged’ and 4.97 for ‘Keeping the session to time’.  Overall, fidelity was high (94.5%; 14.17/15). | | | |
|  | | | |
|  | | | |
|  | | | |
|  | | | |

**Appendix C**

**Participant interview topic guide**

**APPLE-Tree study**

**Attendee interview Topic Guide**

**Introductions:** Thank you for agreeing to take part in this interview. As you know I am a researcher from University College London and I am recording this interview.

**Description of the research:** You have been invited to attend the APPLE-Tree sessions over the past six months. I want to ask you about your experiences of it – the main sessions, the tea breaks, goal calls, catch-up sessions if you received any, and the cognitive training and app if you used them. We are interested in how APPLE-Tree helped you, or not, and for parts you did not use, what might have encouraged you to do so. We will use your suggestions to develop the programme for future participants. There are no right or wrong answers. We would particularly welcome any thoughts about things that could be done differently - please give us your honest opinion as this is what will help us most.

**Q. Please tell me about your experiences of the APPLE-Tree programme.**

**Prompts:**

- - *The initial set-up, being in the group, using zoom*
  - *The difference components: diet, exercise, planning new activities, wellbeing, looking after your mental and physical health*
  - *The tea breaks*
  - *Sending in photos and other material*
  - *Goal calls and setting goals*
  - *Plans to keep in touch with the group/ other participants after the main sessions have needed*
  - *The app*
  - *The cognitive training*

**Q. Were there things about the programme you found helpful?****

**Prompts:**

- - *Can you tell me about that? Have you made any changes to your lifestyle or diet because of the groups? Do you think you will carry on with that change? What might make it easier/ harder to do so?*
  - *Anything else?*
  - *Do you think the group has helped your memory? (If so) Tell me what you have noticed. To what do you attribute that change?*

**Q. Was there anything about the programme you found unhelpful?****

**Prompts:**

- - *Can you tell me about that?*
  - *Do you have any suggestions about how to improve the programme or how it could be different?*
  - *(for participants interviewed because they withdrew): can you tell me about your decision to leave the groups, what influenced your decision?*

****prompt if not discussed for group dynamics, how the group supported each other or not, eg was there a competitive element**

**Q. Before we finish, is there anything else you would like to add?**
